# Prediction of High-Altitude Cardiorespiratory Fitness Impairment Using a Combination of Physiological Parameters During Exercise at Sea Level and Genetic Information in an Integrated Risk Model

**DOI:** 10.3389/fcvm.2021.719776

**Published:** 2022-01-07

**Authors:** Jie Yang, Hu Tan, Mengjia Sun, Renzheng Chen, Jihang Zhang, Chuan Liu, Yuanqi Yang, Xiaohan Ding, Shiyong Yu, Wenzhu Gu, Jingbin Ke, Yang Shen, Chen Zhang, Xubin Gao, Chun Li, Lan Huang

**Affiliations:** ^1^Department of Cardiology, The Second Affiliated Hospital, Third Military Medical University (Army Medical University), Chongqing, China; ^2^Department of Health Care and Geriatrics, The 940th Hospital of Joint Logistics Support Force of PLA, Lanzhou, China; ^3^Department of Ultrasound, Xinqiao Hospital, Army Medical University, Chongqing, China

**Keywords:** cardiorespiratory fitness (CRF), hypoxia, single nucleotide polymorphism, SpO_2_, prediction

## Abstract

Insufficient cardiorespiratory compensation is closely associated with acute hypoxic symptoms and high-altitude (HA) cardiovascular events. To avoid such adverse events, predicting HA cardiorespiratory fitness impairment (HA-CRFi) is clinically important. However, to date, there is insufficient information regarding the prediction of HA-CRFi. In this study, we aimed to formulate a protocol to predict individuals at risk of HA-CRFi. We recruited 246 volunteers who were transported to Lhasa (HA, 3,700 m) from Chengdu (the sea level [SL], <500 m) through an airplane. Physiological parameters at rest and during post-submaximal exercise, as well as cardiorespiratory fitness at HA and SL, were measured. Logistic regression and receiver operating characteristic (ROC) curve analyses were employed to predict HA-CRFi. We analyzed 66 pulmonary vascular function and hypoxia-inducible factor- (HIF-) related polymorphisms associated with HA-CRFi. To increase the prediction accuracy, we used a combination model including physiological parameters and genetic information to predict HA-CRFi. The oxygen saturation (SpO_2_) of post-submaximal exercise at SL and *EPAS1* rs13419896-A and *EGLN1* rs508618-G variants were associated with HA-CRFi (SpO_2_, area under the curve (AUC) = 0.736, cutoff = 95.5%, *p* < 0.001; *EPAS1 A* and *EGLN1 G*, odds ratio [OR] = 12.02, 95% CI = 4.84–29.85, *p* < 0.001). A combination model including the two risk factors—post-submaximal exercise SpO_2_ at SL of <95.5% and the presence of *EPAS1* rs13419896-A and *EGLN1* rs508618-G variants—was significantly more effective and accurate in predicting HA-CRFi (OR = 19.62, 95% CI = 6.42–59.94, *p* < 0.001). Our study employed a combination of genetic information and the physiological parameters of post-submaximal exercise at SL to predict HA-CRFi. Based on the optimized prediction model, our findings could identify individuals at a high risk of HA-CRFi in an early stage and reduce cardiovascular events.

## Introduction

Many people worldwide enjoy mountain hiking at high altitudes (HAs); however, most individuals have never been to a mountain peak and are unaware of the cardiorespiratory fitness requirements for such activities (1). Previous studies showed that hypobaric hypoxia impaired exercise capacity (2) and cardiorespiratory fitness (3, 4), which increased the risk of cardiovascular events in such hypoxic environments (5). Thus, it is crucial to identify individuals who may experience cardiorespiratory fitness impairment (CRFi) before engaging in physical activity at HA.

Cardiorespiratory fitness is a great challenge for those who plan for mountain hiking and an engagement in vigorous physical activity. Previous studies have indicated that cardiorespiratory performance (evaluated by maximum oxygen consumption, VO_2_max) decreases by 1.5–3.5% for every 300 m of additional elevation above 1,500 m (6). This decline is more pronounced at altitudes above 5,000 m (7). Our recent study revealed that right ventricular afterload increase was the major cause of CRFi following acute HA exposure (4). In addition, it has been reported that hypoxic pulmonary vasoconstriction increases pulmonary vascular resistance and decreases pulmonary vascular distensibility (8, 9), as well as contributes to the reduction of aerobic exercise capacity at HA (10). Although HA-induced decreases in arterial oxygenation and pulmonary capillary distensibility are associated with CRFi, the mechanisms that determine this effect in hypoxia remain unknown (11) and little information on predicting CRFi has been reported worldwide.

Pulmonary vascular distensibility is largely limited for most individuals at HA; however, some individuals seem to maintain a reserve that enables them to expand pulmonary vasculature in this environment (12). This may be associated with better maintenance of aerobic exercise capacity at HA. Our previous research also showed that not all individuals experienced CRFi in an acute hypoxic environment (3). The results implied that genetic differences might determine HA cardiorespiratory fitness; however, thus far, little information on this was reported. Therefore, this self-controlled study aimed to analyze 66 polymorphisms in pulmonary vascular function-related (*EDN1, ACE, AGT, PPARA, VEGFA, SLC6A4, NOS3, NFE2L2, CDIP1*, and *ANGPTL4*) and hypoxia-inducible factor- (HIF-) related (*HIF1A, HIF1AN, EGLN3, EGLN1, HMOX2*, and *EPAS1*) pathways and to measure the physiological parameters of post-submaximal exercise both at the sea level (SL) and HA. We hypothesized that a protocol involving genetic information and the parameters of post-submaximal exercise could be formulated to predict individuals at a high risk of HA-CRFi.

## Materials and Methods

### Editorial Policies and Ethical Considerations

This study was registered with the Center of Chinese Clinical Trial Registration (No.: ChiCTR-RCS-12002232), and all procedures were approved by the Clinical Research Ethics Board at the Third Military Medical University (Army Medical University) (identification code: 2012014 approved on May 9, 2012) and conformed to the standards set by the Declaration of Helsinki. All subjects provided a written informed consent.

### Study Population

To avoid a confounding influence of gender, this self-controlled study recruited 246 healthy young Chinese men in Chengdu (<500 m, above the sea level [asl]) according to the inclusion and exclusion criteria. The inclusion criteria included the following: (1) subjects who were born and lived in the SL (<500 m) and (2) men aged from 17 to 35 years old. The exclusion criteria included the following: (1) cardiovascular diseases; (2) pneumonopathy and other respiratory diseases; (3) hematological disease; and (4) HA exposure history in the last 6 months. All subjects provided a written informed consent, were transferred to Lhasa (3,700 m) through an airplane (approximately a 2-h journey), and completed the follow-ups.

### Submaximal Exercise Testing

All subjects underwent a routine medical examination. Then, exercise tests were performed 2 days before the departure at Chengdu (<500 m, asl) and 12 h after the arrival at Lhasa (3,700 m, asl). We recorded the physiological parameters along with submaximal exercise performance. Subjects were told to refrain from the physical exertion 12 h before the exercise test and to refrain from consuming alcohol, caffeine, and tobacco for at least 5 h before the exercise test. To ensure that the exercise test could be conveniently and easily repeated, the subjects performed the exercise for 2 min by stepping up 60 times and stepping down 24 cm (13, 14). We monitored heart rate (HR) and oxygen saturation (SpO_2_) through the finger probe continuously throughout the exercise using the Onyx 9500 (Nonin Medical, Inc., Plymouth, MN, USA), and their precise measurement rate was 2%. The remaining HR and SpO_2_ were measured at the fingertip after at least 10 min of rest, with warm hands, and after 30 s of signal stabilization for an average of the three consecutive measurements before the exercise. While subjects perform post-submaximal exercise, post-submaximal exercise SpO_2_ was measured after the exercise test was terminated. Blood pressure was measured using Omron Hem-6200 (Omron Health Care Ltd., Kyoto, Japan) as soon as the exercise test was completed, and its precise measurement was 3 mmHg. Subjects breathed spontaneously without making any attempt to control the depth or frequency of their respiration. A cardiologist was introduced to guide the process of submaximal exercise test.

### Cardiorespiratory Fitness Measurement

We used a modified two-step physical working capacity test to evaluate cardiorespiratory fitness, which predicted the workload at an HR of 170 beats per min (PWC170) (3, 4, 15–17). The assessment was based on two 5-min physical effort events, where each event was concluded when HR approached 120–170 beats per min. The average HR values were recorded at the end of each 5-min period of physical effort, and this served to determine the strain output in accordance with the PWC170. HR was measured by Omron HEM-6200 (Kyoto, Japan), and its precise measurement was 1 beat. The PWC170 was calculated using the following formula:

PWC170 = W1 + (W2 – W1) [170 – P1/P2 – P1]

where W1 and W2 are the outputs of subsequent physical efforts expressed in watt (W), and P1 and P2 are the HRs measured during a particular physical effort event.

Cardiorespiratory fitness was assessed at both SL and HA. We compared the values of SL and HA cardiorespiratory fitness for all subjects. Compared to SL, the individuals who had a higher cardiorespiratory fitness at SL were classified as the noncardiorespiratory fitness impairment group (non-CRFi), and the individuals with a reduced HA cardiorespiratory fitness were classified as the CRFi group.

### Single-Nucleotide Polymorphism Selection and Genotyping

Altogether, 66 putative single-nucleotide polymorphisms (SNPs) in pulmonary vascular function-related (*EDN1, ACE, AGT, PPARA, VEGFA, SLC6A4, NOS3, NFE2L2, CDIP1*, and *ANGPTL4*) and HIF-related (*HIF1A, HIF1AN, EGLN3, EGLN1, HMOX2*, and *EPAS1*) pathways were selected from the dbSNP database (http://www.ncbi.nlm.nih.gov/SNP) and the genotyping data of the HapMap project (http://www.1000genomes.org/) for an analysis. Information regarding each SNP, including the genotypes, frequency, minor allele frequency (MAF), and the Hardy–Weinberg Equilibrium (HWE), is summarized in Supplementary Table 1. Genomic DNA was extracted from whole blood according to the instructions provided by the AxyPrep Blood Genomic DNA Miniprep kit (Axygen Biosciences, Union City, CA, USA). Genomic DNA samples were stored at −20°C until analysis. PCR primer pairs were designed using the Sequenom Assay Design software (Version 3.1, Sequenom, Inc., San Diego, CA, USA) and synthesized by Sangon Biotech. Genotyping was performed using the MassARRAY MALDI-TOF platform (Sequenom, Inc., San Diego, CA, USA) according to the manufacturer's protocol. The Sequenom Typer 4.0 software was used to perform data management and analysis.

### Covariates

Demographic parameters, namely, age, race, body mass index (BMI), and drinking and smoking history, were recorded. Physiological characteristics, such as systolic blood pressure (SBP) and diastolic blood pressure (DBP), were measured using Omron Hem-6200 (Kyoto, Japan). SpO_2_ was detected with warm hands at the fingertip using a pulse oximeter (Onyx 9500, Nonin Medical, Inc., Plymouth, MN, USA). The measurements were performed after the subjects were given rest for more than 30 min and were repeated more than two times.

### Statistical Analysis

The Kolmogorov–Smirnov test was performed to check for a normal distribution of continuous data. Normally distributed continuous data were expressed as the mean ± SD. Comparisons between the groups of normal distribution were performed using the independent sample *t*-test. A paired-sample *t*-test was employed to compare self-matching data. The median (interquartile range) was used to represent the data that did not meet the criteria for a normal distribution. Mann–Whitney and Wilcoxon signed-rank tests were performed to compare between the groups and self-matching data of abnormal distribution, respectively. A chi-squared test was employed to compare categorical variables between the groups. The sample sizes of all volunteers in this study meet the statistical requirements of an SNP analysis according to the calculation that is dependent on Power and Sample Size Free Online Calculators (http://powerandsamplesize.com).

The prediction accuracy of the SpO_2_ model was expressed using the area under the curve (AUC) of the receiver operating characteristics (ROCs) curve, sensitivity and specificity, and Youden's index (sensitivity + specificity - 1). We obtained the cutoff value according to the maximum Youden's index. Logistic regression of the online software SNPStats (https://www.snpstats.net/start.htm) was applied to investigate and screen the correlation between genotypes and cardiorespiratory fitness. In this analysis, the group with a wild-type genotype was regarded as the reference group. The SPSS version 19.0 statistical package (SPSS, Chicago, IL, USA) was employed to combine the physiological parameters with genotypes to predict cardiorespiratory fitness. Multivariate logistic regression was performed using the covariates including age, race, BMI, and a history of drinking and smoking to adjust these confounding factors. In this analysis, the group with a wide-type genotype and SpO_2_ >95% of post-submaximal exercise was regarded as the reference group. The value of *p* < 0.05 was considered as statistically significant. Genotype and allele distributions, odds ratios (ORs), and 95% CIs were calculated by multivariate logistic regression. The number of subjects in our study conformed to the number size for multivariate logistic regression by adjusting the five confounding factors. The HWE and allele frequencies were confirmed by the SNPStats (https://www.snpstats.net/start.htm) online software.

## Results

### Demographic Characteristics and Physiological Parameters

**Figure 1:**
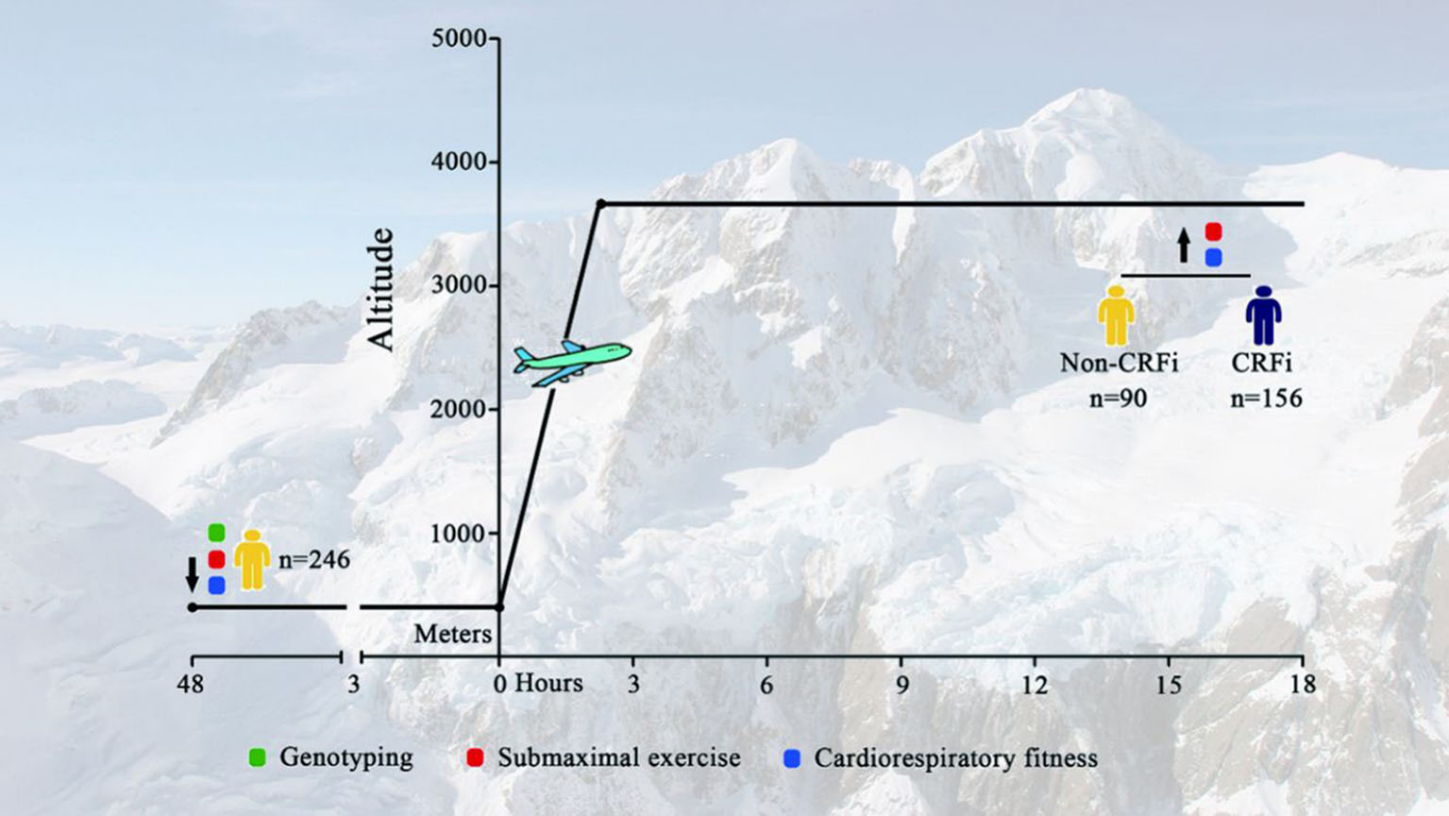
Timeline for assessment. Subjects were transported to Lhasa [HA, 3,700 m above the sea level (asl)] from Chengdu (SL <500 m) through an airplane. Physiological parameters, including SpO_2_, SBP, DBP, and HR, at SL and HA were collected. Submaximal exercise and cardiorespiratory fitness tests were performed at SL and HA (red and blue circles, respectively). Demographic data and genetic information were obtained at SL before departure (green circle). HA, high-altitude; SL, sea level; SpO_2_, oxygen saturation; SBP, systolic blood pressure; DBP, diastolic blood pressure; HR, heart rate; CRF, cardiorespiratory fitness impairment.

A total of 246 healthy young Chinese men were transported to HA (3,700 m, asl) through an airplane (Figure 1). The baseline characteristics of enrolled subjects, stratified by CRFi after acute HA exposure, are reported in Table 1. There was no statistical difference in demographic characteristics in the CRFi and non-CRFi groups. Additional physiological variables at SL, including HR, SpO_2_, SBP, and DBP, are summarized in Table 2.

**Table 1:** Demographic parameters.

**Variables**	**Whole cohort** **(*n* = 246)**	**CRFi** **(*n* = 156, 63.41%)**	**Non-CRFi** **(*n* = 90, 36.59%)**	***P* value**
Age (year)	22 (20–24)	22 (20–24)	22 (20–24)	0.580
Height (cm)	172.00 (169.00–174.00)	170.00 (168.00–173.00)	172.75 (169.00–175.00)	0.271
Weight (kg)	67.00 (62.00–71.00)	65.00 (60.00–69.00)	66.50 (62.00–70.25)	0.512
BMI (kg/m^2^)	22.51 ± 2.16	22.64 ± 2.29	22.29 ± 1.90	0.220
**Ethnicity**				
Han (n, %)	215 (87.40)	137 (87.82)	78 (86.67)	0.843
Non-Han (n, %)	31 (12.60)	19 (12.18)	12 (13.33)	
**Tobacco consumption**				
Yes (n, %)	154 (62.20)	100 (64.10)	54 (60.00)	0.585
No (n, %)	92 (37.80)	56 (35.90)	36 (40.00)	
**Alcohol consumption**				
Yes (n, %)	124 (50.00)	80 (51.28)	44 (48.89)	0.791
No (n, %)	122 (50.00)	76 (48.72)	46 (51.11)	

*CRFi, cardiorespiratory fitness impairment; BMI, body mass index. Values are presented as median (range interquartile). Normally distributed continuous data were expressed as the mean ± SD. The median (interquartile range) was used to represent the data that did not meet the criteria for a normal distribution*.

**Table 2:** Effects of acute HA exposure on BP.

**Variables** **(*n* = 246)**	**SL** **(<500 m, asl)**	**HA** **(3700 m, asl)**	**Post-submaximal** **exercise at SL**	**Post-submaximal** **exercise at HA**	***P_**1**_, P_**2, **_ P_**3**_***
HR (bpm)	65 (61–70)	82 (76–90)	94 (84–105)	137 (128–146)	<0.001 <0.001 <0.001
SpO_2_ (%)	98 (97–99)	88 (87–90)	96 (94–97)	86 (84–89)	<0.001 <0.001 <0.001
SBP (mmHg)	116 (106–123)	115 (108–124)	118 (109–125)	123 (113–131)	0.151 0.018 <0.001
DBP (mmHg)	75 (68–82)	76.50 (69–85)	76.50 (65–85)	75 (70–81)	0.027 0.619 0.118

*SL, sea level; HA, high altitude; HR, heart rate; SBP, systolic blood pressure; DBP, diastolic blood pressure; SpO_2_, oxygen saturation. Values are presented as median (range interquartile), P1 represents the comparison between SL and HA, P2 represents the comparison between SL and after exercise at SL, and P3 represents the comparison between HA and after exercise at HA*.

### Submaximal Exercise and Cardiorespiratory Fitness

As shown in Table 2, HR and DBP increased, but SpO_2_ reduced after acute HA exposure for around 12 h. After submaximal exercise, HR and SBP increased, but SpO_2_ reduced at both SL and HA compared to the baseline levels. Conversely, there was no significant difference in DBP after the exercise at either SL or HA (Table 2).

**Figure 2:**
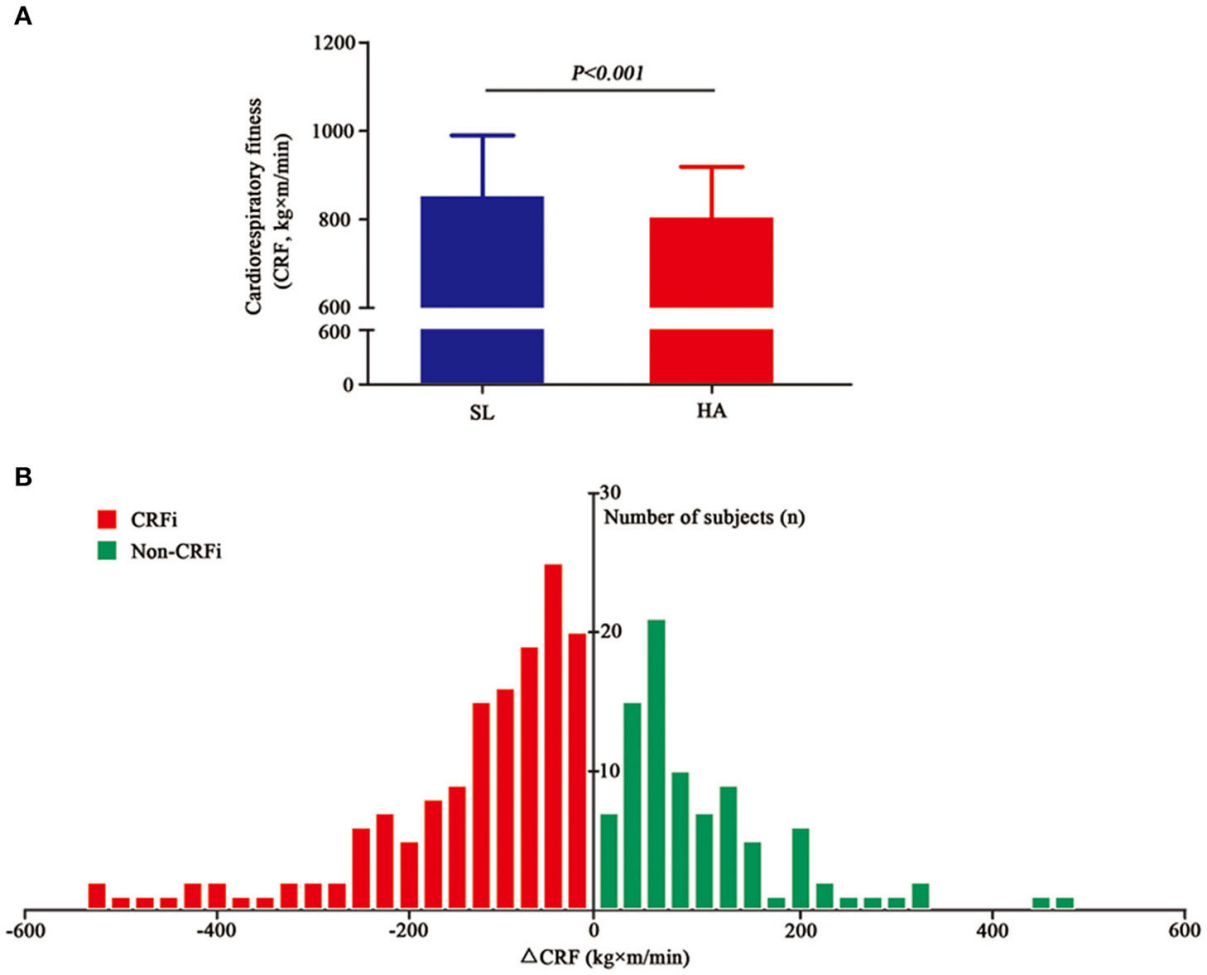
Cardiorespiratory fitness at SL and HA. **(A)** The cardiorespiratory fitness significantly reduced from 829.50 (761.15–908.31) kg×m/min at SL (blue histogram, <500 m) to 798.83 (748.83–821.27) kg×m/min after acute high-altitude exposure (HA) (red histogram, 3,700 m, asl), *p* < 0.001. **(B)** The changes in cardiorespiratory fitness after acute high-altitude exposure. The x-axis represents the values of change in cardiorespiratory fitness. The red histogram represents the number of subjects who experienced CRFi, while the green histogram represents the number of subjects whose cardiorespiratory fitness improved. Values are presented as medians (range interquartile). CRFi, cardiorespiratory fitness impairment; SL, sea level; HA, high altitude; asl, above the sea level.

As shown in Figure 2A and Supplementary Figure 1, cardiorespiratory fitness reduced significantly from 829.50 (761.15–908.31) to 798.83 (748.83–821.27) kg×m/min after acute HA exposure (*p* < 0.001). Although 156 subjects showed impaired CRF, 90 individuals still exhibited an improvement in cardiorespiratory fitness. The data implied that not all individuals experienced CRFi following acute HA exposure. The distribution of cardiorespiratory fitness change is presented in Figure 2B. Furthermore, we grouped subjects into the CRFi group (CRFi, *n* = 156) and the non-impairment group (non-CRFi, *n* = 90) according to the change in cardiorespiratory fitness. The physiological parameters of the two groups are summarized in Table 3. There was no difference in the two groups at HA baseline and post-submaximal exercise. Interestingly, the subjects in the CRFi group showed a lower SpO_2_ compared to those in the non-CRFi group after submaximal exercise at SL, suggesting that, compared to HA, individuals with higher post-submaximal exercise SpO_2_ at SL had better cardiorespiratory fitness.

**Table 3:** Physiological parameters in CRFi and non-impairment groups.

	**Non-CRFi** **(n = 90)**	**CRFi** **(n = 156)**	***P* value**	**Non-CRFi** **(n = 90)**	**CRFi** **(n = 156)**	***P* value**
	**SL**			**HA**		
HR (bpm)	65 (61–69)	65 (61–71)	0.825	84 ± 9	82 ± 10	0.357
SpO_2_ (%)	98 (97–99)	98 (97–99)	0.904	88 (87–90)	89 (87–90)	0.413
SBP (mmHg)	116 (106–124)	116 (106–122)	0.626	115 (108–124)	116 (107–124)	0.667
DBP (mmHg)	75 (68–81)	74 (68–83)	0.893	76.50 (69–85)	75.50 (69–84)	0.236
	**Post–submaximal exercise at SL**			**Post–submaximal exercise at HA**		
HR (bpm)	93 ± 14	97 ± 17	0.064	137 ± 12	138 ± 13	0.403
SpO_2_ (%)	96 (96–98)	95 (94–96)	<0.001	87(84–89)	86 (84–88)	0.215
SBP (mmHg)	118 (109–125)	117 (108–125)	0.097	121 ± 13	122 ± 13	0.527
DBP (mmHg)	74 (64–81)	74 (64–85)	0.118	75 ± 8	76 ± 9	0.340

*SL, sea level; HA, high altitude; non-CRFi, non-cardiorespiratory fitness impairment group; CRFi, cardiorespiratory fitness impairment group; HR, heart rate; SBP, systolic blood pressure, DBP, diastolic blood pressure, MAP, mean arterial pressure, SpO_2_, oxygen saturation. Values are presented as mean ± SD or median (range interquartile)*.

### Regression Analysis of Physiological Parameters With High-Altitude Cardiorespiratory Fitness

**Figure 3:**
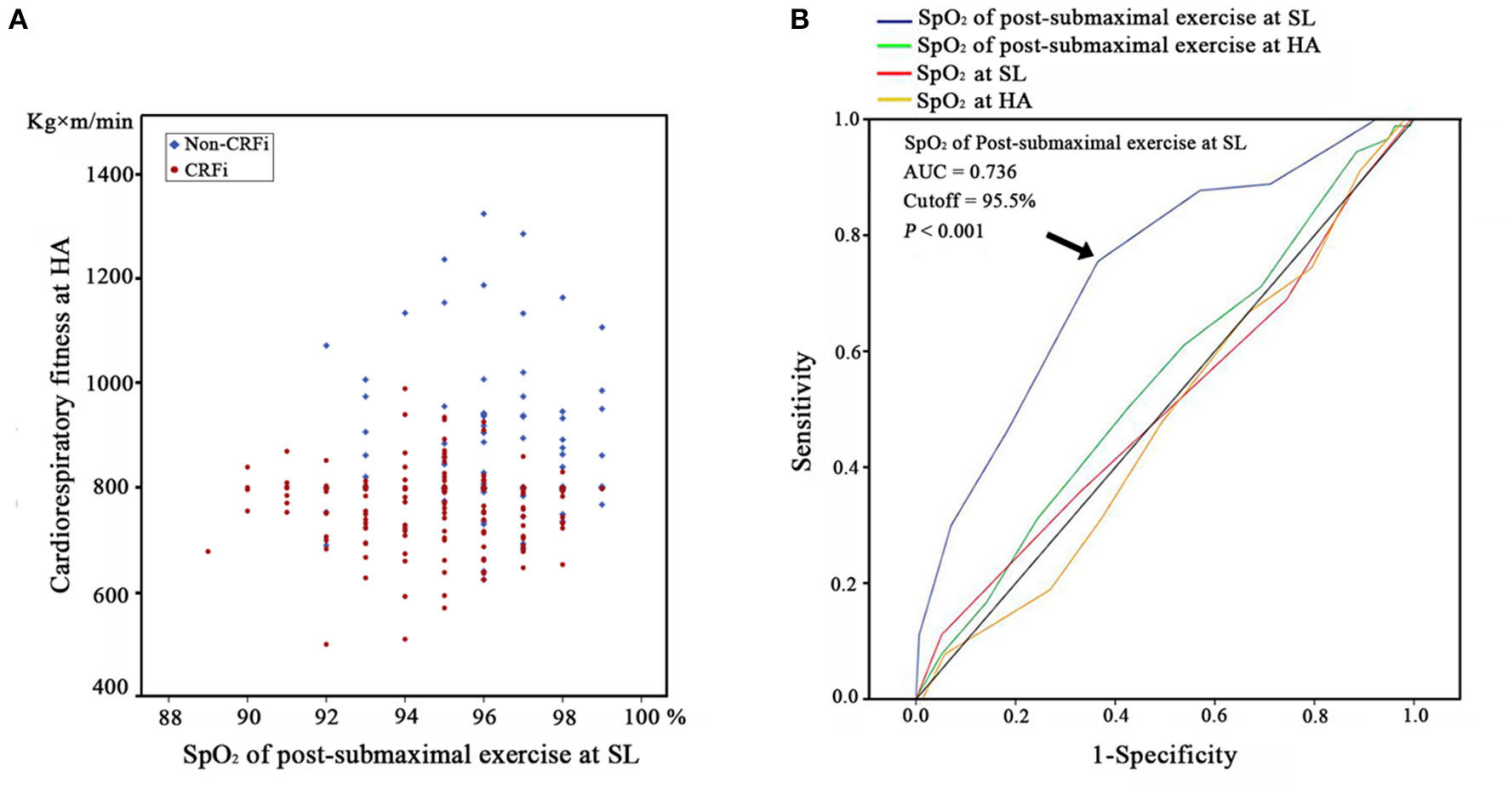
Post-submaximal exercise SpO_2_ at SL predicts the cardiorespiratory fitness at HA. **(A)** The distribution of HA cardiorespiratory fitness according to the different post-submaximal exercise SpO_2_ measurements at SL. The red plots represent the subjects in the CRFi group, while blue plots represent the non-cardiorespiratory fitness impairment (CRFi) group. **(B)** ROC curves and AUCs for post-submaximal exercise SpO_2_ at SL (blue line, AUC = 0.736, 95% CI = 0.6 72–0.801, *p* < 0.001), post-submaximal exercise SpO_2_ at HA (green line, AUC = 0.547, 95% CI = 0.473–0.622, *p* = 0.217), SpO_2_ at SL (red line, AUC = 0.505, 95% CI = 0.428–0.582, *p* = 0.897), and SpO_2_ at HA (yellow line, AUC = 0.478, 95% CI = 0.403–0.552, *p* = 0.560) in the predictive models for CRFi. HA, high altitude; SpO_2_, oxygen saturation; SL, sea level; CRFi, cardiorespiratory fitness impairment; AUC, area under the curve; ROC, receiver operating characteristic.

We found that post-submaximal exercise SpO_2_ at SL (OR_adj_ = 0.619, 95% CI = 0.524–0.730, *p* < 0.001) was closely related to CRFi adjusted for age, BMI, and ethnicity, as well as current smoking and drinking habits (Table 4). The distribution of HA cardiorespiratory fitness for the different SpO_2_ results for post-submaximal exercise at SL is shown in Figure 3A, suggesting that the subjects with higher post-submaximal exercise SpO_2_ at SL may not experience CRFi (blue plots in Figure 3A). A higher post-submaximal exercise SpO_2_ might represent better pulmonary ventilation, oxygen transport, and circulation function. We considered that post-submaximal exercise SpO_2_ at SL was due to the cardiorespiratory fitness reserve capacity following acute hypoxia exposure, which might be a predictive factor. Therefore, we employed a ROC curve based on the maximal Youden's index to find the optimal risk cutoff points. Figure 3B presents the results of the AUC calculations for CRFi at HA according to SpO_2_ levels. Based on the ROC curve analysis, only post-submaximal exercise SpO_2_ at SL displayed a significantly high AUC value for detecting CRFi at HA (AUC = 0.736, *p* < 0.001), and the SpO_2_ cutoff was 95.5%.

**Table 4:** Regression analysis of physiological parameters with high-altitude CRFi.

	**OR (95% CI)**	***P* value**	**OR_**adj**_ (95% CI)**	***P* value**
**SL**				
SBP (mmHg)	1.002 (0.979–1.025)	0.883	0.999 (0.976–1.023)	0.951
DBP (mmHg)	1.001 (0.975–1.027)	0.961	1.000 (0.974–1.027)	0.983
SpO_2_ (%)	0.968 (0.765–1.225)	0.786	0.968 (0.761–1.231)	0.789
**HA**				
SBP (mmHg)	1.005 (0.983–1.026)	0.679	1.002 (0.980–1.024)	0.861
DBP (mmHg)	0.985 (0.960–1.010)	0.229	0.982 (0.958–1.008)	0.172
SpO_2_ (%)	1.058 (0.943–1.187)	0.338	1.072 (0.954–1.204)	0.243
**Post–submaximal exercise at SL**				
SBP (mmHg)	0.982 (0.961–1.005)	0.124	0.981 (0.959–1.004)	0.099
DBP (mmHg)	0.981 (0.959–1.003)	0.087	0.978 (0.956–1.001)	0.058
SpO_2_ (%)	0.626 (0.532–0.736)	<0.001	0.619 (0.524–0.730)	<0.001
**Post-submaximal exercise at HA**				
SBP (mmHg)	1.006 (0.987–1.027)	0.526	1.005 (0.985–1.026)	0.606
DBP (mmHg)	1.015 (0.985–1.046)	0.339	1.013 (0.982–1.045)	0.404
SpO_2_ (%)	0.939 (0.857–1.029)	0.178	0.942 (0.859–1.033)	0.200

*SL, sea level; HA, high altitude; SpO_2_, oxygen saturation; SBP, systolic blood pressure; DBP, diastolic blood pressure; Adjusted for age, BMI, ethnicity, as well as current smoking and drinking. A univariate regression analysis of the physiological parameters with high-altitude cardiorespiratory fitness. The subjects were classified into the non-CRFi and CRFi groups, according to the change in CRF after acute high-altitude exposure*.

### Correlation Between Genotypes and High-Altitude Cardiorespiratory Fitness

We obtained DNA from all the individuals and identified HIF-related and pulmonary vasculature-related SNPs. The genotypes, MAF, and HWE of these SNPs are summarized in Supplementary Table 1. No significant deviations from the HWE were observed for any of the genetic variants.

**Figure 4:**
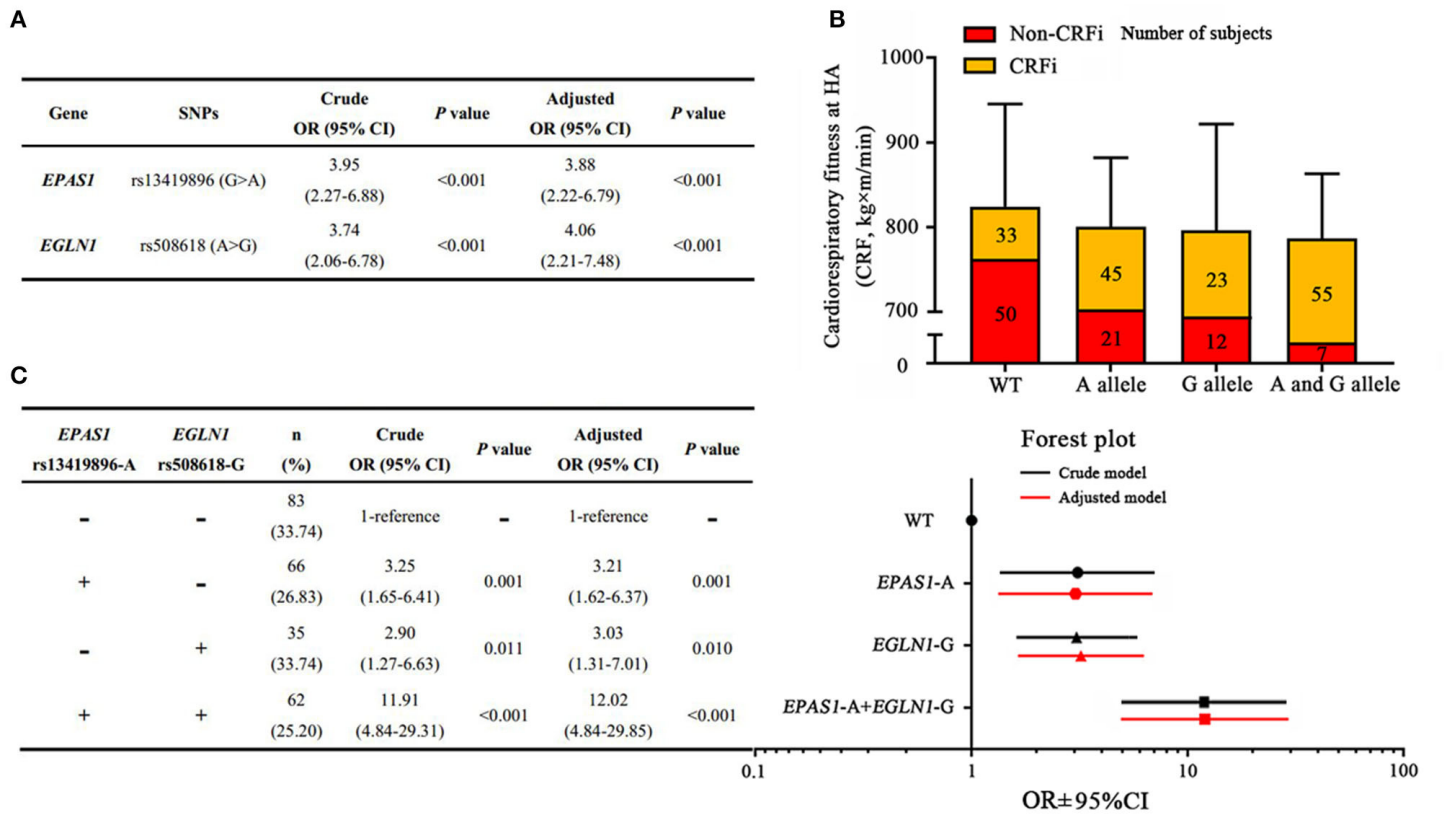
Associations between polygenic variants and risk of high-altitude CRFi. **(A)** The association of *EPAS1* and *EGLN1* with HA cardiorespiratory fitness. **(B)** The distribution of subjects and cardiorespiratory fitness in each group. A total of 246 subjects were divided into four subgroups according to the genotype. Wild type (WT) represents the subjects carrying neither *EPAS1* rs13419896-A nor *EGLN1* rs508618-G, and the cardiorespiratory fitness in this group was 800.83 (798.83–936.41) kg×m/min. The A allele represents the subjects carrying *EPAS1* rs13419896-A but not *EGLN1* rs508618-G, and the cardiorespiratory fitness in this group was 798.83 (730.64–907.20) kg×m/min. The G allele represents the subjects carrying *EGLN1* rs508618-G but not *EPAS1* rs13419896-A, and the cardiorespiratory fitness in this group was 797.05 (735.75–800.83) kg×m/min. The A and G alleles represent the subjects carrying both *EPAS1* rs13419896-A and *EGLN1* rs508618-G, and the cardiorespiratory fitness in this group was 782.46 (683.16–810.00) kg×m/min. There was no significant difference between the groups at HA. The yellow and red squares represent the proportion of CRFi and non-CRFi in each subgroup. The number of subjects in each subgroup is shown on the histogram. **(C)** The association of the four subgroups with HA cardiorespiratory fitness. The forest plot shows the OR and 95% CI of subgroups compared to the WT in the adjusted model. The x-axis of the forest plot was numbered using log10 in GraphPad prism 7.0. WT, wild type; SNP, single nucleotide polymorphism; CRFi, cardiorespiratory fitness impairment; OR, odds ratio; 95% CI, 95% CI; HA, high altitude. Adjusted for age, BMI, ethnicity, as well as current smoking and drinking habits.

We assessed the correlation between these SNPs and the cardiorespiratory fitness changes following acute HA exposure in an adjusted model. The data are shown in Supplementary Table 2. Among these SNPs, logistic regression analysis revealed that the HIF-related genes *EPAS1* and *EGLN1* were associated with CRFi. The rs13419896-A allele mutation in *EPAS1* significantly increased the risk of CRFi (OR_adj_ = 3.88, 95% CI = 2.22–6.79, *p* < 0.001), while the rs508618-G allele mutation in *EGLN1* remarkably increased the impairment (OR_adj_ = 4.06, 95% CI = 2.21–7.48, *p* < 0.001) after an adjustment for confounders (Figure 4A).

### Synergistic Effect of Polygenic Variants on High-Altitude Cardiorespiratory Fitness

As *EPAS1* and *EGLN1* variants were found to be independent risk factors for CRFi, we evaluated the synergistic effect of polygenic variations. Subjects were divided into four subgroups according to genotype. The distribution of subjects and cardiorespiratory fitness in each group are shown in Figure 4B. In carriers of both *EPAS1* rs13419896-A allele and *EGLN1* rs508618-G allele variants, the risk of CRFi increased almost 12-fold compared to those carrying a wild type after an adjustment for confounders (OR_adj_ = 12.02, 95% CI = 4.84–29.85, *p* < 0.001). In *EGLN1* rs508618-G allele non-carriers, *EPAS1* rs13419896-A allele variation was associated with a 3.21-fold increased risk of CRFi (OR_adj_ = 3.21, 95% CI = 1.62–6.37, *p* = 0.001). The subjects carrying the *EGLN1* rs508618-G allele without the *EPAS1* rs13419896-A allele also showed an increased risk of CRFi (OR_adj_ = 3.03, 95% CI = 1.31–7.01, *p* = 0.010) (Figure 4C).

### High-Altitude Fitness Prediction

**Figure 5:**
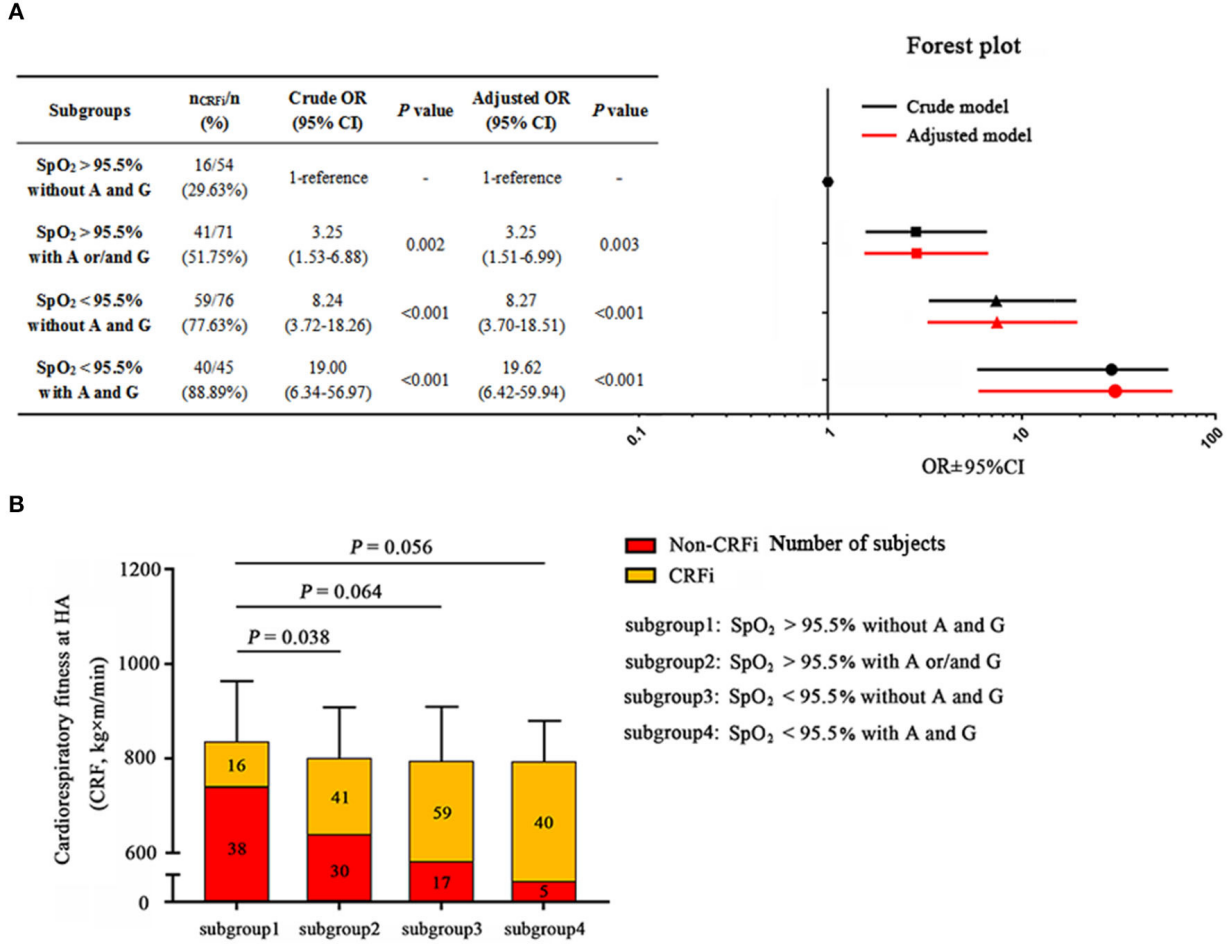
The Combination model prediction of high-altitude cardiorespiratory fitness. **(A)** Subjects are divided into subgroups: subgroup 1, post-submaximal exercise SpO_2_ at SL >95.5% and carrying neither the *EPAS1* rs13419896-A nor *EGLN1* rs508618-G; subgroup 2, post-submaximal exercise SpO_2_ at SL >95.5% and carrying *EPAS1* rs13419896-A and/or *EGLN1* rs508618-G; subgroup 3, post-submaximal exercise SpO_2_ at SL <95.5% and not carrying *EPAS1* rs13419896-A or *EGLN1* rs508618-G; subgroup 4, post-submaximal exercise SpO_2_ at SL <95.5% and carrying *EPAS1* rs13419896-A and *EGLN1* rs508618-G. Post-submaximal exercise SpO_2_ at SL <95.5% combined with carrying *EPAS1* rs13419896-A and *EGLN1* rs508618-G was the most effective prediction model with OR = 19.62 and 95% CI: 6.42–59.94, *p* < 0.001. The forest plot shows the OR and 95% CI of subgroups compared to the WT in the adjusted model. The x-axis of the forest plot was numbered by log10 in GraphPad prism 7.0. **(B)** The distribution of subjects and cardiorespiratory fitness in the four subgroups. The cardiorespiratory fitness of subgroup 1 to subgroup 4 was 834.59 ± 131.08, 800.27 ± 109.89, 793.62 ± 117.34, and 793.24 ± 88.67 kg×m/min, respectively. Subgroups 2 (*p* = 0.038), 3 (*p* = 0.064), and 4 (*p* = 0.056) had lower cardiorespiratory fitness compared to subgroup 1. The yellow and red squares represent the proportion of CRFi and non-CRFi in each subgroup, respectively. The number of subjects in each subgroup is shown on the histogram. SpO_2_, oxygen saturation; SL, sea level; HA, high altitude; CRFi, cardiorespiratory fitness impairment; OR, odds ratio; 95% CI, 95% CI. Adjusted for age, BMI, and ethnicity, as well as current smoking and drinking habits.

As both post-submaximal exercise SpO_2_ and *EPAS1* and *EGLN1* variants were closely related to HA cardiorespiratory fitness, we constructed a combined predictive model according to the optimal SpO_2_ cutoff and risk factors for the *EPAS1* A and/or *EGLN1* G alleles. The largest OR increment was observed when we combined the two risk factors: SpO_2_ <95.5% together with carrying both *EPAS1* A and *EGLN1* G alleles. The subjects with an SpO_2_ of <95.5% after submaximal exercise at SL and who carried *EPAS1* A and *EGLN1* G alleles had a greater than 19-fold (95% CI: 6.42–59.94, *p* < 0.001) increased risk of CRFi at HA after an adjustment for confounders compared to individuals in the wild-type group (Figure 5A). Although the other two predictive models were effective and significant, this combined predictive model was more accurate. It was superior to the models involving carrying the A and/or G alleles with SpO_2_ >95.5% after post-maximal exercise (OR = 3.25, 95% CI: 1.51–6.99, *p* = 0.003) and SpO_2_ <95.5% after post-maximal exercise without risk alleles (OR = 8.27, 95% CI: 3.70–18.51, *p* < 0.001).

When we divided the cohort into four subgroups according to the models, HA cardiorespiratory fitness was lower in the three risk subgroups compared to the wild-type group. Furthermore, the incidence of CRFi in the combined predictive model was the highest (almost 90%, Figure 5B) among the four subgroups. This optimized predictive model provides an effective and accurate method to identify the individuals who may be at risk of CRFi at HA and to direct them to improve their physical performance.

## Discussion

This self-controlled study is the first to predict HA cardiorespiratory fitness based on the information obtained at SL. The most noteworthy finding was that the combination of information regarding post-submaximal exercise SpO_2_ at and genetic information significantly improved the prediction of HA cardiorespiratory fitness impairment (HA-CRFi), compared to the use of SpO_2_ alone or genetic information alone. Our results provide a new strategy to identify individuals at risk of HA-CRFi before participation in physical activity at HA.

Cardiorespiratory fitness was confirmed as a powerful predictor of chronic disease morbidity and mortality half a century ago (18). Prospective observational studies have shown that low levels of cardiorespiratory fitness are strongly associated with the risk of developing cardiovascular diseases (6, 19, 20), hypertension (21, 22), diabetes mellitus (23, 24), cancers (25–27), and all-cause mortality (28, 29). It is imperative to evaluate cardiorespiratory fitness for the prevention, early detection, or diagnosis of potential health issues among the susceptible population. At present, regular physical exercise, especially aerobic exercise, is considered as the most effective and convenient method for improving cardiorespiratory fitness in the normoxia condition. Previous reports have indicated that body composition and race will affect fitness (30), and thus we adjust the confounders, including ethnicity and BMI, in this study. Cardiorespiratory fitness involves multiorgan synergistical works, including oxygen uptake, exchange and transport, and circulatory activity, as well as myocardium and skeletal muscle oxygenation. The altitude-induced reduction in both convective oxygen transfer and systemic oxygen availability provides a great challenge in maintaining cardiorespiratory fitness.

Interestingly, previous studies indicated a reduction of HA cardiorespiratory fitness by about 10% (6). However, in our study, an overall decrease in cardiorespiratory fitness was 4%, approximately, compared to previous research. Several factors may be associated with the difference. Firstly, all the subjects that we enrolled in this study were healthy young Chinese men, who had a better cardiorespiratory fitness tolerance. Moreover, this difference may be associated with the altitude reached and the method of cardiorespiratory fitness measurement. Besides, the way and the time of HA exposure were also contributed to this difference. Our previous study revealed that the altitude-induced decline of cardiorespiratory fitness was related to decreased SpO_2_ and elevated mean pulmonary arterial pressure (31). This study further revealed that the genetic profile determined a variation of cardiorespiratory fitness in acute hypoxic exposure and post-submaximal exercise SpO_2_ at SL might predict HA cardiorespiratory fitness.

Although *EPAS1* and *EGLN1* variants synergistically increased the risk of HA-CRFi, the association of the two genes with SpO_2_ is still unclear. Accordingly, we compared the SpO_2_ at rest and after submaximal exercise in the four subgroups as shown in Supplementary Figure 2. The baseline SpO_2_ at SL showed no significant difference among the four subgroups, which indicated that the two variants have a little influence on the SpO_2_ in the normoxic condition. The subjects carrying *EGLN1* rs508618-G variants exhibited lower SpO_2_ with acute HA exposure. Both of the two variant carriers showed significantly lower SpO_2_ after submaximal exercise compared to the wild type and carriers with only one risk variant. These results implied that *EPAS1* and *EGLN1* synergistically contributed to SpO_2_ reduction after submaximal exercise.

The *EPAS1* gene encodes HIF2α, a subunit of the HIF complex, which involves regulating the response to hypoxia. Conversely, *EGLN1* encodes hydroxylase domain protein 2, the principal negative regulator of HIFs. Previous population-based genetic research studies have confirmed that *EGLN1* and *EPAS1* variants show a positive natural selection in highland natives (3, 32). These mutations were closely related to phenotypic variations of hemoglobin concentration in Tibetans, which facilitated an adaptation to the hypoxic environment (23, 33). Besides maintaining hemoglobin concentration through the regulation of EPO, *EPAS1* and *EGLN1* are associated with a series of pathological and physiological processes (24). *EPAS1* contains 16 exons extending more than 90 kb on 2p21–p16, with a large intron 1 (50 kb) (25). Our recent research found that rs6756667 wild-type GG homozygous genotype in *EPAS1* elevated the risk of having acute mountain sickness (26). In addition, *EPAS1* is important for cancer progression (27), angiogenesis (29), pulmonary hypertension (30), and the progression of chronic obstructive pulmonary disease (COPD) (34). The A allele at rs13419896 of *EPAS1* is associated with an enhanced expression and prognosis for non-small cell lung cancer (27). Rs13419896 is localized at the region of intron 1. The bioinformatic analysis suggested that the area including rs13419896 contained transcriptional regulatory elements, which played a crucial role in regulating the *EPAS1* gene expression. We found that the G allele at rs13419896 of *EPAS1* was associated with a higher risk of impaired cardiorespiratory fitness than the variants lacking the G allele. We speculated that the nucleotide difference at the rs13419896 locus may affect the function of enhancer activities that further maintain *EPAS1* expression and activate HIF pathways. On the other hand, the genetic polymorphisms of *EGLN1* have been commonly reported to be related to disease development, such as tumor aggressiveness (35), erythrocytosis (36–38), AMS (39, 40), and HA pulmonary edema (41). Most of this research focused on the effect of different variants on *EGLN1* expression and HIF-1α regulation. Our results suggested that *EPAS1* acted in concert with *EGLN1* to orchestrate the transcriptional response to hypoxia and HA cardiorespiratory fitness.

## Limitations

This study has some limitations. Firstly, the sample size in this self-controlled study is not very large, and consequently, large-scale, multi-center, and prospective research studies are highly warranted in the future. Secondly, both the maximal aerobic capacity test (VO_2_max test) and physical work capacity test (PWC170 test) were employed to assess cardiorespiratory fitness according to previous reports (4, 14, 39). In this study, we used an indirect measurement of the PWC170 test to evaluate cardiorespiratory fitness due to HA and ethical considerations. VO_2_max tests require sophisticated equipment and a maximal effort on the part of the subject. Alternatively, the PWC170 test can be conducted with minimal cost and equipment, and the subjects do not require a maximal effort, which can be performed conveniently and safely in the hypobaric hypoxic environment. Thirdly, as only Chinese young men were recruited in this self-controlled study, and the genetic polymorphism between men and women was significantly different, our prediction model was mostly applied to young men. Finally, we chose a finger pulse oximeter instead of a blood gas analyzer to measure SpO_2_ for its convenience and safety. To apply this model to women and all age groups, we need to further recruit more volunteers to evaluate the validity of this model in the near future.

## Conclusion

To date, most genomic screenings and association studies have focused on the differences in hypobaric hypoxic acclimatization and metabolism pathways between highland natives and lowlanders, and a few studies have investigated HA aerobic performance and cardiorespiratory fitness. Our study is the first to predict HA-CRFi depending on the physiological parameters at SL and genetic information. Our findings provide a practical and clinical strategy to identify individuals at risk of HA cardiorespiratory fitness before attempting the physical exertion at HA. Adverse cardiorespiratory events can be avoided in people wanting to undertake physical insertion at HA.

## Data Availability Statement

The datasets presented in this study can be found in online repositories. The names of the repository/repositories and accession number(s) can be found in the article/Supplementary Material.

## Ethics Statement

This study was registered with the Center of Chinese Clinical Trial Registration (No: ChiCTR-RCS-12002232), and all procedures were approved by the Clinical Research Ethics Board at the Third Military Medical University (Army Medical University) (identification code: 2012014). The patients/participants provided their written informed consent to participate in this study.

## Author Contributions

LH conceived and supervised this self-controlled study. JY, SY, JZ, CLiu, RC, YY, and XG followed all subjects both at the sea level and high altitude and performed the genetic information as well as collected the physiological parameters. JY guided subjects in performing sub-maximal exercise both at the sea level and high altitude. The genetic analysis was conducted by JY and CLiu. The sequence data analysis and statistics were made by CZ, YS, WG, MS, and YY. The manuscript was written by JY and HT. LH reviewed and revised this manuscript. All authors approved the final version of manuscript.

## Funding

This study was supported by the National Natural Science Foundation of China (Grant Nos. 81800396 and 81730054), the Chongqing Talents: Exceptional Young Talents Project, and Chongqing Natural Science Foundation (cstc2019jcyj-msxmX0471).

## Conflict of Interest

The authors declare that the research was conducted in the absence of any commercial or financial relationships that could be construed as a potential conflict of interest.

## Publisher's Note

All claims expressed in this article are solely those of the authors and do not necessarily represent those of their affiliated organizations, or those of the publisher, the editors and the reviewers. Any product that may be evaluated in this article, or claim that may be made by its manufacturer, is not guaranteed or endorsed by the publisher.

## Supplementary Material

Supplementary Table 1 Genotype frequencies.

Supplementary Table 2 Association of genetic variants and risk on cardiorespiratory fitness impairment in acute hypoxia exposure.

Supplementary Figure 1 Cardiorespiratory fitness in total, the cardiorespiratory fitness impairment (CRFi) and non-cardiorespiratory fitness impairment (non-CRFi) groups. The cardiorespiratory fitness significantly reduced in total from 829.50 (761.15–908.31) kg×m/min at the sea level (SL, red) to 798.83 (748.83–821.27) kg×m/min after acute high-altitude exposure (red), *p* < 0.001. In the CRFi group, cardiorespiratory fitness significantly reduced from 864.62 (817.51–962.56) to 795.4 (723.14–801.63) kg×m/min after acute high-altitude exposure (green), *p* < 0.001. In the non-CRFi group, cardiorespiratory fitness significantly reduced from 760.8 (727.74–798.88) to 801.8 (798.87–937.87) kg×m/min after acute high-altitude exposure (blue), *p* < 0.001.

Supplementary Figure 2 The SpO_2_ for different variant carriers at HA and SL. **(A)** There was no significant difference in the baseline SpO_2_ in the four subgroups. **(B)** The SpO_2_ of *EGLN1* rs508618-G variant carriers (87 [86–88]%) was significantly lower than that in the WT group (89 [87–91]%, *p* < 0.01) and *EPAS1* rs13419896-A variant carriers (89 [87–90.25]%, *p* < 0.01), as well as in the two variant carriers (88 [86.75–90.25]%, *p* < 0.01) at HA. **(C)** The subjects carrying both the *EGLN1* rs508618-G and *EPAS1* rs13419896-A variants (96 [95–97]%) showed remarkably lower post-submaximal exercise SpO_2_ at SL in comparison to the WT carriers (96 [95–97]%, *p* < 0.01), the *EPAS1* rs13419896-A variant carriers (95 [94–96]%, *p* < 0.01), and the *EGLN1* rs508618-G variant carriers (96 [95–97]%, *p* < 0.01). **(D)** The subjects carrying both the *EGLN1* rs508618-G and *EPAS1* rs13419896-A variants (86 [85–88]%, *p* < 0.01) showed significantly lower post-submaximal exercise SpO_2_ at HA compared to WT carriers (89 [86–91]%, *p* < 0.01), *EPAS1* rs13419896-A variant carriers (89 [87–91]%, *p* < 0.01), and *EGLN1* rs508618-G variant carriers (88 [87–91]%, *p* < 0.01). SpO_2_, oxygen saturation; HA, high-altitude; SL, sea level; WT, wild type.
