# Supplementary material for: Prediction of High-Altitude Cardiorespiratory Fitness Impairment Using a Combination of Physiological Parameters During Exercise at Sea Level and Genetic Information in an Integrated Risk Model
Source: Front Cardiovasc Med. 2022 Jan 7;8:719776. doi: 10.3389/fcvm.2021.719776 (PMC8782201; doi:10.3389/fcvm.2021.719776)
Supplement: Supplementary Table 1 — Genotype frequencies. [file Table_1.DOCX]

Supplementary Material

**Supplementary Table S1. Genotype frequencies.**

| Gene | SNPs | Genotypes | Frequency cases | MAF | | *P*-HWE |
| --- | --- | --- | --- | --- | --- | --- |
| *ACE* | rs1055086 | G/G | 77 | 0.47 | | 0.068 |
|  |  | A/G | 106 |  |  |  |
|  |  | A/A | 60 |  |  |  |
|  | rs4329 | G/G | 89 | 0.40 | | 0.997 |
|  |  | A/G | 119 |  |  |  |
|  |  | A/A | 38 |  |  |  |
|  | rs4353 | A/A | 48 | 0.45 | | 0.976 |
|  |  | A/G | 123 |  |  |  |
|  |  | G/G | 75 |  |  |  |
|  | rs4461142 | C/C | 75 | 0.46 | | 0.496 |
|  |  | C/T | 114 |  |  |  |
|  |  | T/T | 53 |  |  |  |
|  | rs8066114 | C/C | 144 | 0.23 | | 0.626 |
|  |  | C/G | 91 |  |  |  |
|  |  | G/G | 11 |  |  |  |
|  | rs4291 | A/A | 78 | 0.43 | | 0.568 |
|  |  | A/T | 126 |  |  |  |
|  |  | T/T | 42 |  |  |  |
| *AGT* | rs699 | C/C | 174 | 0.16 | | 1.00 |
|  |  | C/T | 66 |  |  |  |
|  |  | T/T | 6 |  |  |  |
| *ANGPTL4* | rs4076317 | C/C | 17 | 0.26 | | 0.87 |
|  |  | C/G | 91 |  |  |  |
|  |  | G/G | 128 |  |  |  |
| *CDIP1* | rs1051308 | A/A | 118 | 0.29 | | 0.64 |
|  |  | A/G | 94 |  |  |  |
|  |  | G/G | 22 |  |  |  |
| *EDN1* | rs2070699 | T/T | 68 | 0.48 | | 0.70 |
|  |  | G/T | 119 |  |  |  |
|  |  | G/G | 58 |  |  |  |
|  | rs2248580 | A/A | 85 | 0.42 | | 0.36 |
|  |  | A/C | 112 |  |  |  |
|  |  | C/C | 48 |  |  |  |
|  | rs5370 | G/G | 133 | 0.27 | | 0.10 |
|  |  | G/T | 85 |  |  |  |
|  |  | T/T | 23 |  |  |  |
| *EGLN1* | rs12406290 | G/G | 60 | 0.48 | | 0.25 |
|  |  | A/G | 112 |  |  |  |
|  |  | A/A | 71 |  |  |  |
|  | rs12757362 | G/G | 244 | 0.004 | | 1.00 |
|  |  | C/G | 2 |  |  |  |
|  | rs1339891 | G/G | 214 | 0.07 | | 1.00 |
|  |  | A/G | 31 |  |  |  |
|  |  | A/A | 1 |  |  |  |
|  | rs1339894 | G/G | 246 | - | | - |
|  | rs1361384 | A/A | 240 | 0.004 | | 1.00 |
|  |  | A/G | 2 |  |  |  |
|  | rs1538667 | A/A | 232 | 0.03 | | 1.00 |
|  |  | A/G | 14 |  |  |  |
|  | rs2009873 | A/A | 96 | 0.37 | | 0.89 |
|  |  | A/G | 116 |  |  |  |
|  |  | G/G | 33 |  |  |  |
|  | rs2066140 | G/G | 92 | 0.38 | | 0.59 |
|  |  | C/G | 121 |  |  |  |
|  |  | C/C | 33 |  |  |  |
|  | rs2153364 | G/G | 63 | 0.48 | | 0.095 |
|  |  | A/G | 108 |  |  |  |
|  |  | A/A | 72 |  |  |  |
|  | rs2275279 | A/A | 138 | 0.26 | | 0.51 |
|  |  | A/T | 90 |  |  |  |
|  |  | T/T | 18 |  |  |  |
|  | rs2486729 | A/A | 98 | 0.38 | | 0.18 |
|  |  | A/G | 105 |  |  |  |
|  |  | G/G | 41 |  |  |  |
|  | rs2739513 | A/A | 95 | 0.37 | | 0.78 |
|  |  | A/G | 114 |  |  |  |
|  |  | G/G | 31 |  |  |  |
|  | rs2808609 | T/T | 232 | 0.03 | | 1.00 |
|  |  | C/T | 14 |  |  |  |
|  | rs7542797 | A/A | 199 | 0.10 | | 0.46 |
|  |  | A/C | 40 |  |  |  |
|  |  | C/C | 3 |  |  |  |
|  | rs508618 | A/A | 149 | 0.23 | | 0.47 |
|  |  | A/G | 82 |  |  |  |
|  |  | G/G | 15 |  |  |  |
|  | rs2486736 | A/A | 93 | 0.38 | | 0.79 |
|  |  | A/G | 119 |  |  |  |
|  |  | G/G | 34 |  |  |  |
| *EGLN3* | rs11156819 | C/C | 127 | 0.29 | | 0.22 |
|  |  | C/T | 93 |  |  |  |
|  |  | T/T | 25 |  |  |  |
|  | rs1680710 | G/G | 231 | 0.02 | | 1.00 |
|  |  | A/G | 11 |  |  |  |
| *EPAS1* | rs6756667 | G/G | 189 | 0.13 | | 0.56 |
|  |  | A/G | 52 |  |  |  |
|  |  | A/A | 5 |  |  |  |
|  | rs4953354 | A/A | 180 | 0.18 | | 0.83 |
|  |  | A/G | 58 |  |  |  |
|  |  | G/G | 8 |  |  |  |
|  | rs1868092 | A/A | 8 | 0.14 | | 0.11 |
|  |  | A/G | 53 |  |  |  |
|  |  | G/G | 185 |  |  |  |
|  | rs13419896 | A/A | 24 | 0.31 | | 0.88 |
|  |  | A/G | 104 |  |  |  |
|  |  | G/G | 118 |  |  |  |
| *HIF1AN* | rs2295778 | C/C | 153 | 0.22 | | 0.26 |
|  |  | C/G | 78 |  |  |  |
|  |  | G/G | 15 |  |  |  |
|  | rs1054399 | C/C | 205 | 0.08 | | 0.39 |
|  |  | C/T | 41 |  |  |  |
|  | rs11190613 | T/T | 205 | 0.08 | | 0.39 |
|  |  | C/T | 41 |  |  |  |
|  | rs11816840 | G/G | 205 | 0.08 | | 0.39 |
|  |  | C/G | 41 |  |  |  |
|  | rs3750633 | G/G | 205 | 0.08 | | 0.39 |
|  |  | A/G | 41 |  |  |  |
|  | rs11292 | T/T | 205 | 0.08 | | 0.39 |
|  |  | C/T | 41 |  |  |  |
|  | rs11190602 | C/C | 3 | 0.11 | | 1.00 |
|  |  | C/T | 49 |  |  |  |
|  |  | T/T | 194 |  |  |  |
| *HIF1A* | rs12434438 | A/A | 115 | 0.30 | 0.093 | |
|  |  | A/G | 115 |  |  |  |
|  |  | G/G | 16 |  |  |  |
|  | rs2301113 | A/A | 210 | 0.08 | | 1.00 |
|  |  | A/C | 35 |  |  |  |
|  |  | C/C | 1 |  |  |  |
|  | rs2301112 | A/A | 210 | 0.07 | | 1.00 |
|  |  | A/C | 35 |  |  |  |
|  |  | C/C | 1 |  |  |  |
|  | rs2301104 | G/G | 477 | 0.03 | | 1.00 |
|  |  | C/G | 15 |  |  |  |
|  | rs966824 | C/C | 140 | 0.23 | | 0.15 |
|  |  | C/T | 96 |  |  |  |
|  |  | T/T | 9 |  |  |  |
|  | rs11549467 | G/G | 228 | 0.04 | | 1.00 |
|  |  | A/G | 18 |  |  |  |
| *HMOX2* | rs9921781 | C/C | 246 | - | | - |
| *NOS3* | rs1799983 | G/G | 206 | 0.09 | | 0.69 |
|  |  | G/T | 38 |  |  |  |
|  |  | T/T | 2 |  |  |  |
| *PPARA* | rs4253623 | A/A | 172 | 0.17 | | 0.64 |
|  |  | A/G | 66 |  |  |  |
|  |  | G/G | 8 |  |  |  |
|  | rs4253681 | T/T | 160 | 0.19 | | 0.40 |
|  |  | C/T | 80 |  |  |  |
|  |  | C/C | 6 |  |  |  |
|  | rs4253747 | T/T | 153 | 0.21 | | 0.44 |
|  |  | A/T | 85 |  |  |  |
|  |  | A/A | 8 |  |  |  |
|  | rs7292407 | A/A | 13 | 0.19 | | 0.09 |
|  |  | A/C | 66 |  |  |  |
|  |  | C/C | 107 |  |  |  |
|  | rs6520015 | C/C | 13 | 0.20 | | 0.16 |
|  |  | C/T | 71 |  |  |  |
|  |  | T/T | 162 |  |  |  |
|  | rs135538 | C/C | 48 | 0.48 | | 0.057 |
|  |  | C/G | 138 |  |  |  |
|  |  | G/G | 60 |  |  |  |
| *SLC6A4* | rs1042173 | G/G | 179 | 0.14 | | 0.43 |
|  |  | G/T | 64 |  |  |  |
|  |  | T/T | 3 |  |  |  |
|  | rs3813034 | C/C | 145 | 0.25 | | 0.058 |
|  |  | C/A | 80 |  |  |  |
|  |  | A/A | 21 |  |  |  |
|  | rs7224199 | T/T | 179 | 0.15 | | 0.8 |
|  |  | G/T | 61 |  |  |  |
|  |  | G/G | 6 |  |  |  |
| *VEGFA* | rs10434 | G/G | 134 | 0.25 | | 0.31 |
|  |  | A/G | 100 |  |  |  |
|  |  | A/A | 12 |  |  |  |
|  | rs3025039 | C/C | 178 | 0.14 | | 0.44 |
|  |  | C/T | 65 |  |  |  |
|  |  | T/T | 3 |  |  |  |
|  | rs3025040 | C/C | 179 | 0.14 | | 0.43 |
|  |  | C/T | 64 |  |  |  |
|  |  | T/T | 3 |  |  |  |
|  | rs1413711 | A/A | 17 | 0.30 | | 0.13 |
|  |  | A/G | 114 |  |  |  |
|  |  | G/G | 115 |  |  |  |
| *NFE2L2* | rs10497511 | C/C | 21 | 0.29 | | 0.88 |
|  |  | C/T | 100 |  |  |  |
|  |  | T/T | 125 |  |  |  |
|  | rs6721961 | G/G | 125 | 0.30 | | 0.17 |
|  |  | G/T | 94 |  |  |  |
|  |  | T/T | 27 |  |  |  |
|  | rs1962142 | C/C | 143 | 0.24 | | 0.60 |
|  |  | C/T | 87 |  |  |  |
|  |  | T/T | 16 |  |  |  |
|  | rs2364722 | A/A | 59 | 0.48 | | 0.70 |
|  |  | A/G | 120 |  |  |  |
|  |  | G/G | 67 |  |  |  |

SNP, single nucleotide polymorphism; MAF, minor allele frequency; HWE, Hardy-Weinberg Equilibrium.

**Supplementary Table S2. Association of genetic variants and risk on cardiorespiratory fitness impairment in acute hypoxia exposure.**

| **Genes** | **SNPs** | **Crude**  **OR 95%CI** | ***P* value** | **Adjusted**  **OR 95 %CI** | ***P* value** |
| --- | --- | --- | --- | --- | --- |
| ***ACE*** | rs1055086 (G > A) | 1.04 (0.59 -1.82) | 0.89 | 1.05 (0.60-1.85) | 0.86 |
|  | rs4329 (G > A) | 0.82 (0.36-1.71) | 0.48 | 0.83 (0.48-1.44) | 0.51 |
|  | rs4353 (G > A) | 0.89 (0.50-1.71) | 0.68 | 0.91 (0.51-1.61) | 0.74 |
|  | rs4461142 (C > T) | 1.00 (0.36-1.24) | 0.99 | 1.04 (0.58-1.85) | 0.90 |
|  | rs8066114 (C > G) | 0.95 (0.56-1.61) | 0.85 | 0.99 (0.58-1.69) | 0.96 |
|  | rs4291 (A > G) | 0.81 (0.46-1.43) | 0.47 | 0.81 (0.46-1.43) | 0.46 |
| ***AGT*** | rs699 (C > T) | 0.87 (0.49-1.53) | 0.63 | 0.80 (0.44-1.43) | 0.44 |
| ***ANGPTL4*** | rs4076317 (G > C) | 0.74 (0.43-1.25) | 0.26 | 0.68 (0.40-1.17) | 0.16 |
| ***COIP1*** | rs1051308 (A > G) | 1.17 (0.69-2.00) | 0.56 | 1.27 (0.74-2.18) | 0.39 |
| ***EDN1*** | rs2070699 (T > G) | 1.22 (0.69-2.17) | 0.72 | 1.23 (0.68-2.21) | 0.49 |
|  | rs2248580 (A > C) | 1.09 (0.63-1.88) | 0.75 | 1.06 (0.61-1.85) | 0.83 |
|  | rs5370 (G > T) | 1.28 (0.76-2.18) | 0.35 | 1.29 (0.75-2.22) | 0.35 |
| ***EGLN1*** | rs12406290 (G > A) | 1.40 (0.80-2.47) | 0.24 | 1.43 (0.80-2.56) | 0.22 |
|  | rs1339891 (G > A) | 1.86 (0.80-4.34) | 0.13 | 1.91 (0.81-4.48) | 0.12 |
|  | rs1275362 (G > C) | 0.57 (0.04-9.29) | 0.70 | 0.51 (0.03-8.55) | 0.64 |
|  | rs1339894 (GG) | monomorphic SNP | | | |
|  | rs1361384 (A > G) | NA (0.00-NA) | 0.18 | NA (0.00-NA) | 0.16 |
|  | rs1538667 (A > G) | 1.04 (0.34-3.21) | 0.94 | 1.16 (0.36-3.72) | 0.80 |
|  | rs2009873 (A > G) | 0.91 (0.53-1.55) | 0.73 | 0.91 (0.53-1.57) | 0.74 |
|  | rs2066140 (G > C) | 0.88 (0.52-1.51) | 0.65 | 0.90 (0.52-1.56) | 0.70 |
|  | rs2153364 (G > A) | 1.32 (0.75-2.32) | 0.33 | 1.37 (0.77-2.43) | 0.29 |
|  | rs2275279 (A > T) | 0.84 (0.50-1.41) | 0.51 | 0.85 (0.50-1.45) | 0.55 |
|  | rs2486729 (A > G) | 0.88 (0.52-1.50) | 0.64 | 0.89 (0.51-1.53) | 0.66 |
|  | rs2486736 (A > G) | 0.86 (0.50-1.47) | 0.58 | 0.88 (0.51-1.52) | 0.63 |
|  | rs2739513 (A > G) | 0.87 (0.51-1.49) | 0.62 | 0.87 (0.50-1.51) | 0.62 |
|  | rs2808609 (T > C) | 1.04(0.34-3.21) | 0.94 | 1.16 (0.36-3.72) | 0.80 |
|  | rs7542797 (A > C) | 1.40 (0.69-2.84) | 0.35 | 1.54 (0.75-3.18) | 0.23 |
| ***EGLN3*** | rs11156819 (C > T) | 0.96 (0.57-1.61) | 0.86 | 0.94 (0.55-1.60) | 0.82 |
|  | rs1680710 (G > A) | 0.47 (0.14-1.58) | 0.22 | 0.52 (0.15-1.83) | 0.31 |
| ***EPAS1*** | rs6756667 (G > A) | 0.99 (0.53-1.82) | 0.96 | 0.95 (0.51-1.79) | 0.88 |
|  | rs1868092 (G > A) | 0.71 (0.39-1.29) | 0.26 | 0.65 (0.36-1.20) | 0.17 |
|  | rs4953354 (A > G) | 0.85 (0.49-1.48) | 0.58 | 0.80 (0.46-1.41) | 0.45 |
| ***HIF1A*** | rs12434438 (A > G) | 1.23 (0.73-2.07) | 0.44 | 1.23 (0.72-2.09) | 0.45 |
|  | rs2301104 (G > C) | 1.16 (0.39-3.52) | 0.79 | 1.08 (0.35-3.35) | 0.89 |
|  | rs2301112 (A > C) | 0.69 (0.38-1.44) | 0.33 | 0.67 (0.31-1.43) | 0.30 |
|  | rs2301113 (A > C) | 0.68 (0.33-1.39) | 0.29 | 0.66 (0.31-1.38) | 0.27 |
|  | rs966824 (C > T) | 1.61 (0.95-2.75) | 0.077 | 1.53 (0.89-2.63) | 0.12 |
|  | rs11549467 (G > A) | 0.43 (0.16-1.14) | 0.089 | 0.43 (0.16-1.15) | 0.093 |
| ***HIF1AN*** | 11190602 (T > C) | 1.24 (0.65-2.37) | 0.51 | 1.25 (0.64-2.40) | 0.51 |
|  | rs2295778 (C > G) | 1.26 (0.73-2.15) | 0.41 | 1.26 (0.73-2.17) | 0.42 |
|  | rs1054399 (C > T) | 1.48 (0.72-3.08) | 0.28 | 1.47 (0.70-3.08) | 0.30 |
|  | rs3750633 (G > A) | 1.48 (0.72-3.08) | 0.28 | 1.47 (0.70-3.08) | 0.30 |
|  | rs11190613 (T > C) | 1.48 (0.72-3.08) | 0.28 | 1.47 (0.70-3.08) | 0.30 |
|  | rs11292 (T > C) | 1.48 (0.72-3.08) | 0.28 | 1.47 (0.70-3.08) | 0.30 |
|  | rs11816840 (G > C) | 1.48 (0.72-3.08) | 0.28 | 1.47 (0.70-3.08) | 0.30 |
| ***HMOX2*** | rs9921781 (CC) | monomorphic SNP | | | |
| ***NOS3*** | rs1799983 (G > T) | 1.24 (0.60-2.55) | 0.56 | 1.26 (0.61-2.63) | 0.53 |
| ***PPARA*** | rs4253623(A > G) | 1.69 (0.94-3.05) | 0.076 | 1.63 (0.90-2.97) | 0.10 |
|  | rs4253681(T > C) | 0.71 (0.41-1.21) | 0.21 | 0.70 (0.40-1.20) | 0.19 |
|  | rs4253747 (T > A) | 0.69 (0.41-1.18) | 0.18 | 0.71 (0.42-1.22) | 0.21 |
|  | rs135538 (G > C) | 1.46 (0.81-2.64) | 0.22 | 1.38 (0.75-2.52) | 0.30 |
|  | rs6520015 (T > C) | 1.06 (0.61-1.83) | 0.84 | 1.10 (0.63-1.93) | 0.74 |
|  | rs7292407 (C > A) | 1.08 (0.62-1.88) | 0.80 | 1.10 (0.62-1.95) | 0.74 |
| ***SLC6A4*** | rs1042173 (G > T) | 1.66 (0.90-3.06) | 0.097 | 1.67 (0.90-3.10) | 0.095 |
|  | rs7224199 (T > G) | 1.66 (0.90-3.06) | 0.097 | 1.67 (0.90-3.10) | 0.095 |
|  | rs3813034 (C > A) | 1.44 (0.84-2.45) | 0.18 | 1.47 (0.85-2.52) | 0.16 |
| ***VEGFA*** | rs10434 (G > A) | 0.87 (0.52-1.46) | 0.59 | 0.87 (0.51-1.48) | 0.62 |
|  | rs1413711 (G > A) | 1.23 (0.73-2.07) | 0.44 | 1.17 (0.69-1.98) | 0.56 |
|  | rs3025039 (C > T) | 0.83 (0.47-1.48) | 0.53 | 0.85 (0.47-1.51) | 0.57 |
|  | rs3025040 (C > T) | 0.96 (0.54-1.71) | 0.88 | 0.96 (0.54-1.73) | 0.90 |
| ***NFE2L2*** | rs10497511 (T > C) | 0.83 (0.49-1.39) | 0.47 | 0.86 (0.51-1.46) | 0.59 |
|  | rs1962142 (C > T) | 0.85 (0.50-1.43) | 0.53 | 0.90 (0.53-1.53) | 0.70 |
|  | rs2364722 (G > A) | 0.96 (0.53-1.71) | 0.88 | 1.02 (0.56-1.84) | 0.95 |
|  | rs6721961 (G > A) | 0.83 (0.49-1.39) | 0.47 | 0.86 (0.51-1.46) | 0.59 |

SNP: single nucleotide polymorphism; OR: odds ratio; 95% CI: 95% confidence interval; Adjusted for age, current smoking, current drinking, height and weight; p<0.05 indicates statistical significance.
