# Supplementary figures and images for: Prediction of High-Altitude Cardiorespiratory Fitness Impairment Using a Combination of Physiological Parameters During Exercise at Sea Level and Genetic Information in an Integrated Risk Model
Source: Front Cardiovasc Med. 2022 Jan 7;8:719776. doi: 10.3389/fcvm.2021.719776 (PMC8782201; doi:10.3389/fcvm.2021.719776)

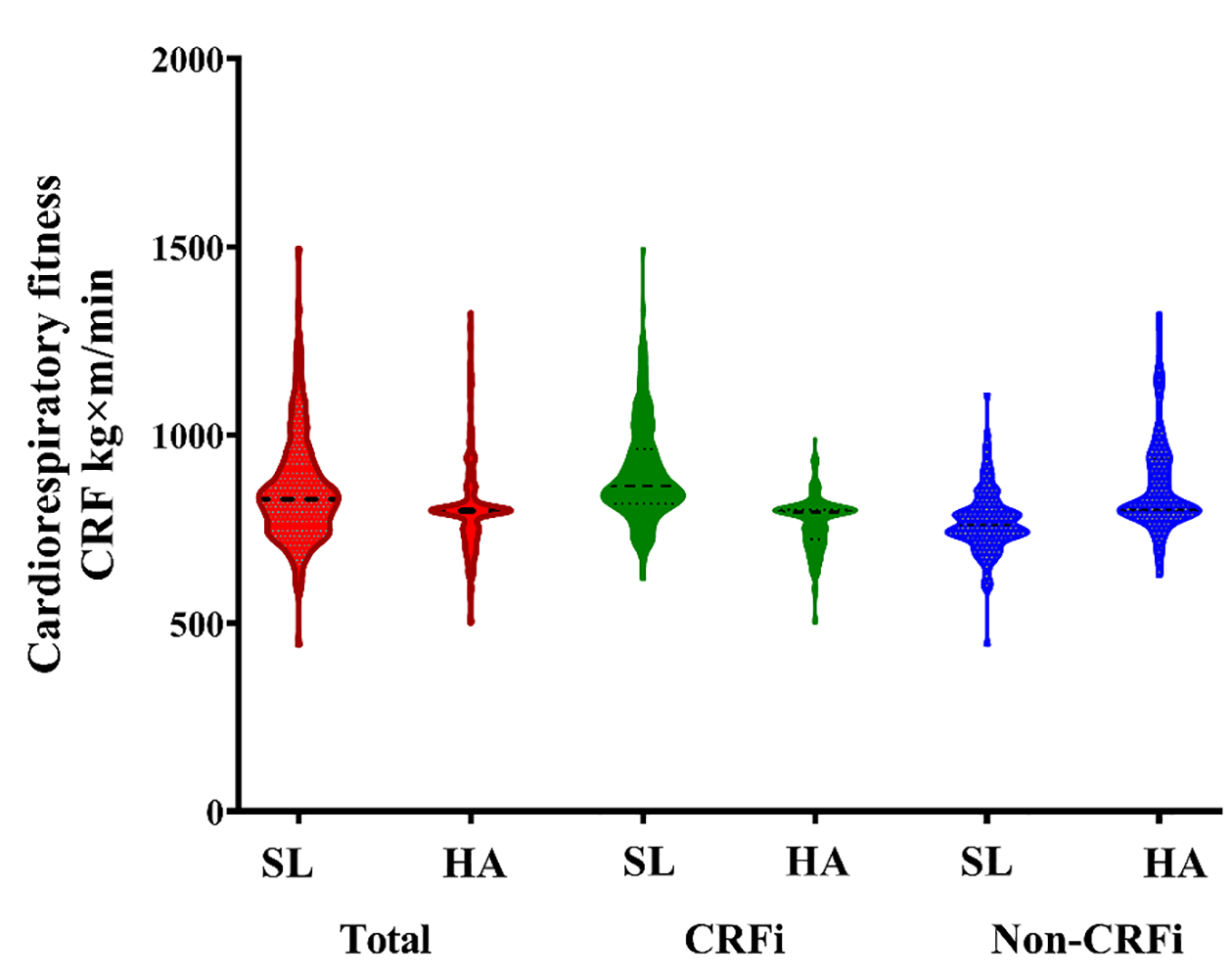

Supplement: Supplementary Figure 1 — Cardiorespiratory fitness in total, the cardiorespiratory fitness impairment (CRFi) and non-cardiorespiratory fitness impairment (non-CRFi) groups. The cardiorespiratory fitness significantly reduced in total from 829.50 (761.15–908.31) kg×m/min at the sea level (SL, red) to 798.83 (748.83–821.27) kg×m/min after acute high-altitude exposure (red), p < 0.001. In the CRFi group, cardiorespiratory fitness significantly reduced from 864.62 (817.51–962.56) to 795.4 (723.14–801.63) kg×m/min after acute high-altitude exposure (green), p < 0.001. In the non-CRFi group, cardiorespiratory fitness significantly reduced from 760.8 (727.74–798.88) to 801.8 (798.87–937.87) kg×m/min after acute high-altitude exposure (blue), p < 0.001. [file Image_1.tif]

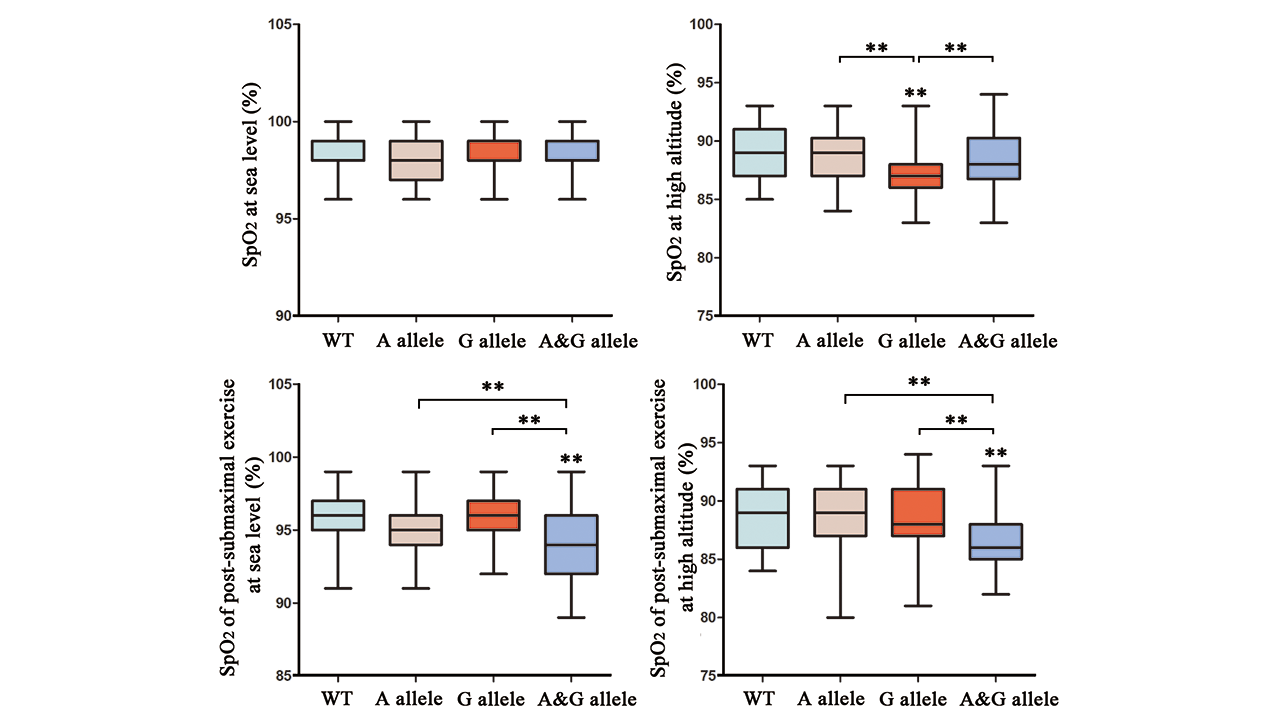

Supplement: Supplementary Figure 2 — The SpO2 for different variant carriers at HA and SL. (A) There was no significant difference in the baseline SpO2 in the four subgroups. (B) The SpO2 of EGLN1 rs508618-G variant carriers (87 [86–88]%) was significantly lower than that in the WT group (89 [87–91]%, p < 0.01) and EPAS1 rs13419896-A variant carriers (89 [87–90.25]%, p < 0.01), as well as in the two variant carriers (88 [86.75–90.25]%, p < 0.01) at HA. (C) The subjects carrying both the EGLN1 rs508618-G and EPAS1 rs13419896-A variants (96 [95–97]%) showed remarkably lower post-submaximal exercise SpO2 at SL in comparison to the WT carriers (96 [95–97]%, p < 0.01), the EPAS1 rs13419896-A variant carriers (95 [94–96]%, p < 0.01), and the EGLN1 rs508618-G variant carriers (96 [95–97]%, p < 0.01). (D) The subjects carrying both the EGLN1 rs508618-G and EPAS1 rs13419896-A variants (86 [85–88]%, p < 0.01) showed significantly lower post-submaximal exercise SpO2 at HA compared to WT carriers (89 [86–91]%, p < 0.01), EPAS1 rs13419896-A variant carriers (89 [87–91]%, p < 0.01), and EGLN1 rs508618-G variant carriers (88 [87–91]%, p < 0.01). SpO2, oxygen saturation; HA, high-altitude; SL, sea level; WT, wild type. [file Image_2.TIF]
